# Ethical practice in my work: community health workers’ perspectives using photovoice in Wakiso district, Uganda

**DOI:** 10.1186/s12910-020-00505-2

**Published:** 2020-08-03

**Authors:** David Musoke, Charles Ssemugabo, Rawlance Ndejjo, Sassy Molyneux, Elizabeth Ekirapa-Kiracho

**Affiliations:** 1 Department of Disease Control and Environmental Health, School of Public Health, College of Health Sciences, Makerere University, Kampala, Uganda; 2 Kenya Medical Research Institute (KEMRI) - Wellcome Trust Research Programme, Kilifi, Kenya; 3 Department of Health Policy, Planning and Management, School of Public Health, College of Health Sciences, Makerere University, Kampala, Uganda

**Keywords:** Ethics, Ethical principles, Ethical challenges, Community health workers, Practice, Photovoice, Uganda

## Abstract

**Background:**

Health service delivery should ensure ethical principles are observed at all levels of healthcare. Working towards this goal requires understanding the ethics-related priorities and concerns in the day-to-day activities among different health practitioners. These practitioners include community health workers (CHWs) who are involved in healthcare delivery in communities in many low-and middle-income countries such as Uganda. In this study, we used photovoice, an innovative community based participatory research method that uses photography, to examine CHWs' perspectives on ethical concerns in their work.

**Methods:**

We explored perspectives of 10 CHWs (5 females and 5 males) on ethical dimensions of their work for 5 months using photovoice in a rural community in Wakiso district, Uganda. As part of the study, we: 1. Oriented CHWs on photovoice research and ethics; 2. Asked CHWs to take photographs of key ethical dimensions of their work; 3. Held monthly meetings to discuss and reflect on the photos; and 4. Disseminated the findings. The discussions from the monthly meetings were audio recorded, transcribed, and emerging data analysed using conventional content analysis with the help of Atlas ti version 6.0.15.

**Results:**

CHWs were aware of and highly concerned about the need to observe ethical principles while carrying out their roles. The ethical principles CHWs were aware of and endeavoured to observe during their work were: maintaining professional integrity and abiding by ethical principles of practice; ethical responsibility in patient care; maintaining confidentiality while handling clients; respect for persons and communities; and enhancing their knowledge and skills for better practice. However, CHWs also identified challenges concerning their observance of ethical principles including: low commitment to their work due to other obligations; availability of some reference materials and guidelines in English yet majority could only read in the local language; and minimal avenues for knowledge enhancement such as trainings.

**Conclusions:**

CHWs were aware of and keen to discuss ethical issues in their work. However, there is need to address the challenges they face so as to facilitate observing ethical principles during the course of their work in communities.

## Background

Community health workers (CHWs) play an important role in supporting health systems especially in providing a range of primary health care services [1, 2]. In low- and middle-income countries (LMICs), CHWs have contributed significantly to reducing undernutrition, improving maternal and child health, expanding access to family planning services, and contributing to control of HIV, malaria and tuberculosis for many years [3]. In Uganda, CHWs locally referred to as Village Health Teams (VHTs), were introduced in 2001 and are the first point of contact of the community with the health system [4, 5]. CHWs in the country are selected by the community based on their ability to read and write preferably in the local language, and their integrity [4–7]. Before commencement of their work, CHWs are mandated to undergo an orientation training that may last approximately 5 days, with refresher trainings on specific topics carried out whenever the need arises [5]. CHWs in Uganda are volunteers but may receive financial and non-financial incentives such as t-shirts, bicycles and bags from the Ministry of Health and other partners that support them [5]. The CHWs are charged with several roles and responsibilities including health education, community mobilisation for public health interventions such as immunisation, disease surveillance, household visits to promote sanitation and hygiene, treatment of children below 5 years of age under integrated community case management (iCCM) of childhood illnesses, and referral of patients to health facilities [4, 5, 8, 9]. Activities carried out by CHWs are community based, and they usually report to health practitioners at health facilities in their area who are responsible for supervising them. Whereas CHWs exist throughout the country, they are known to operate more in rural parts of the country where there is limited access to health facilities [4]. In such rural communities, CHWs have been instrumental in increasing health service delivery particularly for maternal and child health as well as communicable diseases such as malaria.

Ethics are “ethos” or “way of life” or “social norms for conduct” that distinguish between acceptable and unacceptable behaviour [10]. Codes of conduct have long been a strong pillar in health professions, outlining the social norms, rules and responsibilities, and proper practices, of practitioners [11]. A review of the ethical framework for CHWs and related cadres showed that ethical principles include: equal and substantial respect; justice; care; beneficence; community and cultural humility; openness; critical reflection; trustworthiness; and competency [11]. These principles provide practical guidance not only for the work of CHWs but could also be applied to other cadres of the health workforce within national health systems. Given that CHWs are involved in health service delivery, observance of ethical standards during the course of their work is of paramount importance for better health outcomes. Despite the important role of ethical principles in the work of CHWs, little is known about ethical concerns while carrying out their duties including through using visual participatory research methodologies such as photovoice.

Photovoice uses photography to enable people capture and showcase concerns in their communities [12]. The use of photovoice has the potential to assist researchers to work with community members to identify issues that affect their day-to-day lives which can be shared by various stakeholders including policy makers to effect change [12, 13]. The photographs taken during photovoice studies are mainly used to generate discussion among participants as well as for dissemination among various stakeholders including members of the community. Several benefits of using photovoice in research have been identified including community empowerment, capacity building of participants, co-learning between researchers and participants, and enhancing community utilisation of findings [14–16]. Photovoice has previously been used to specifically hear the ideas of those whose voices may be excluded in some contexts including women, people with disabilities, and youth. In addition, participants of photovoice research are able to share their experiences captured on camera with researchers, community members and policy makers [16, 17].

Photovoice can be used in health systems research to generate culturally relevant knowledge that addresses injustice, inequality and exploitation in communities including among CHWs. Understanding perspectives of CHWs on ethics is important in exploring how principles are experienced in their work, including challenges faced. Such an understanding can facilitate in identifying training needs and support processes for CHWs. Our study examined CHWs perspectives on ethical concerns and how they affect their work within the community in rural Wakiso district, Uganda using photovoice.

## Methods

### Study design

The qualitative study, conducted in 2015/16, used photovoice, a community-based participatory research method, and involved 10 CHWs (5 females and 5 males). The participants took photographs on ethical concerns in their community for a period of 5 months, as outlined in detail below. During the course of the study, five monthly meetings involving both participants and research team members were held to discuss the photos which were sufficient to reach data saturation. Indeed, by the end of the fifth monthly meeting, no new emerging issues were emanating from the photos taken by participants and ensuing discussion. This paper focuses on the findings on ethical concerns as other results emerging from the study have been published separately [18].

### Study area, setting and selection of participants

The study was conducted in Bulwanyi, a rural parish in Wakiso district, Uganda. Wakiso is the largest district in the country per population, and is comprised of a mix of urban, peri-urban and rural communities. The main economic activity in Bulwanyi is agriculture, with others being brick making, and small-scale trade. The parish has five villages with each having four CHWs that support the health system in the area. Among the four CHWs per village, two are specifically involved in iCCM in addition to other roles and responsibilities. The other two CHWs only carry out activities such as health education and community mobilisation. Being a rural area, CHWs are the first contact of the community to the health system, and are a link with the one public health facility nearby.

Local leaders including local council members and community mobilisers were involved in selecting the 10 CHWs who were the research participants. These local leaders were considered suitable in selecting participants given their good knowledge of the CHWs in their area. In addition, a good rapport had earlier been established with these local leaders as part of the many projects that the research team had implemented in the study community in previous years. During the selection of participants, it was ensured that CHWs came from different villages as well as had various demographic characteristics such as age and level of education (Table 1). In addition, both CHWs involved and not involved in iCCM were selected to take part in the study. This nature of selection of participants was to ensure diversity in the photos taken during the study and resulting discussions. Once the participants had been selected, the researchers verified that the required criteria had been met.

**Table 1:** Study participant demographics

Participant number	Gender	Age range (years)	Education level	Occupation	Marital status and number of children
1.	Male	50–60	Primary	Agriculture	Married, 8 children
2.	Female	30–40	Secondary level (ordinary)	Agriculture	Married, 5 children
3.	Female	40–50	Secondary level (ordinary)	Agriculture	Widowed, 7 children
4.	Male	40–50	Secondary level (ordinary)	Agriculture	Married, 8 children
5.	Male	40–50	Secondary level (ordinary)	Agriculture	Married, 5 children
6.	Female	40–50	Secondary level (ordinary)	Agriculture	Married, 7 children
7.	Female	50–60	Secondary level (ordinary)	Agriculture	Married, 5 children
8.	Female	20–30	Secondary level (ordinary)	Business	Married, 4 children
9.	Male	20–30	Secondary level (advanced)	Agriculture	Single, no children
10.	Male	30–40	Secondary level (advanced)	Agriculture	Married, 3 children

### Training workshop and photography

A training workshop facilitated by the researchers was held with participants at a project office in the study community. The one-day workshop was to orient the participants on the study including research principles such as voluntary participation, ethics and use of cameras as well as on the use of photovoice in research. The workshop also discussed issues on ethical principles among CHWs to ensure they appreciated the study focus before commencement of photography. The ethical issues discussed in the workshop included respect, trustworthiness, competence, openness, humility and justice. In addition to a camera, each participant was given a notebook for use in situations where photos could not be taken such as when a community member did not consent to photography. The cameras were to be used by participants to take as many photos as they could concerning ethical concerns during the course of their day-to-day work for 5 months. During the course of the study, the researchers worked closely with participants to discuss if and how the photos related to ethical concerns. This included routine visits to the field by the research team to assess progress of the participants and offer any necessary support. More details on the workshop and photography assignment can be found in our earlier paper [18].

### Discussing photographs, data analysis, and community dissemination

During the study, five monthly meetings, each lasting approximately 3 h, were held with participants and researchers at the project office in the study community. During the meetings, participants presented and discussed photos taken in the previous month. The meetings were facilitated by two members of the research team (DM, PhD - moderator and CS, MPH – note taker) who are researchers with vast experience and interest in qualitative research including photovoice. To enhance discussion, a projector was used to display the photos taken by the participants. After a participant had presented each photo they had taken and related it to the study theme, other members had the opportunity to make comments and contribute to the discussion. For participants who had used their notebooks, they had the opportunity to present the issues captured therein to the group after exhausting all their photos. The note taker (CS) was involved in audio recording and taking notes of the discussions during the monthly meetings. The recordings, which were in the local language (*Luganda*), were later transcribed verbatim. Translation to English was done by the research team after verification of the transcripts. Data analysis was carried out by DM and CS using conventional content analysis in Atlas ti version 6.0.15. During data analysis, codes arising from the data were identified which formed categories and then themes as noted in more detail elsewhere [18]. The major themes on ethical concerns from the analysis are presented in this paper as well as selected photographs and quotations from participants. After the five monthly meetings, a community dissemination event was held to share the major findings from the study to various stakeholders. This meeting was attended by CHWs, local leaders, community mobilisers, health authorities, and community members. During the dissemination meeting, photos selected jointly by the researchers and CHWs were presented by the CHWs. It was important to involve the participants in selection of photos for the dissemination to ensure those most relevant to their community were included. The CHWs talked about the photos and explained to the audience how they related to the community. After the presentation of photos, the audience asked questions and made contributions regarding how the findings could be used to improve health in the community. For example, it was suggested that the local leaders needed to work closely with CHWs to ensure community members are aware of and support the work of these health volunteers.

### Ethical consideration

The research was approved by Makerere University School of Public Health Higher Degrees, Research and Ethics committee (protocol 304), and the Uganda National Council for Science and Technology (SS 3848). During the training workshop, participants were explained to all aspects of the research including potential risks and benefits before they voluntarily provided written informed consent. Participants were to receive verbal consent from community members before they took their photographs as part of the research. However, this was not always possible and at times challenging for example in scenarios where there was no sufficient time to obtain such consent. In such situations, participants used their notebooks to record the issue of concern, or devised means of taking photos in line with ethical principles for example without people’s faces being identifiable in them. Before using any photo from the research for dissemination such as conference presentation and publication, written consent of both the photographer and any person appearing in it was obtained.

## Results

During the 5 months of the study, 432 photographs were taken which included those on ethical concerns presented in this paper. Discussions of photographs on ethical concerns during the meetings showed that CHWs were aware of the need for ethical practice in the course of their day-to-day work. In addition, the CHWs were highly concerned about the need to observe ethical principles while carrying out their roles. The ethical practices highlighted by the CHWs that emerged from the data analysed from the monthly discussions are presented under five themes: maintaining professional integrity and abiding by ethical principles of practice; ethical responsibility in patient care; maintaining confidentiality while handling clients; respect for persons and communities; and enhancing knowledge and skills. The final theme presented in this paper provides some of the practical challenges that CHWs face during their work which undermine their ethical practice.

### Maintaining professional integrity and abiding by ethical principles of practice

CHWs stated that it was their responsibility to maintain professional integrity and abide by ethical principles of practice. The CHWs highlighted the need to espouse honesty and truthfulness in their work while dealing with clients. In addition, the CHWs said honesty was required in communicating potential benefits and consequences of available services, and that they should be truthful about their competences and limitations on the services they provide. Indeed, the CHWs noted the importance of acknowledging when client issues are outside their scope of practice, and referring them to the health facility as noted below:

*“I was called when this man had eye problems after dust had entered them. Since I couldn’t handle that condition, I advised him to go to the nearby health facility where skilled health practitioners would be able to treat that condition.”* CHW 9, male

CHWs also highlighted the need to be committed to their work, and perform their roles and responsibilities diligently. For example, the CHWs said that they identify health challenges in the community such as disease outbreaks, poor health seeking behaviour, unsanitary practices, occupational health concerns, and teenage pregnancies. The CHWs noted that after problem identification, they took steps to solve them for example by educating community members about the existence of the problem, mobilising them to take action or notifying concerned health authorities.

*“That child was spraying pesticides in the garden without wearing any personal protective equipment. I first explained to him the importance of using personal protective equipment when handling harmful chemicals. Thereafter, I advised him to obtain and wear gloves, masks and gumboots when spraying those chemicals. I emphasized that non-use of protective gear could result in inhalation and / or ingestion of chemicals.”* CHW 4, male

**Fig. 1:**
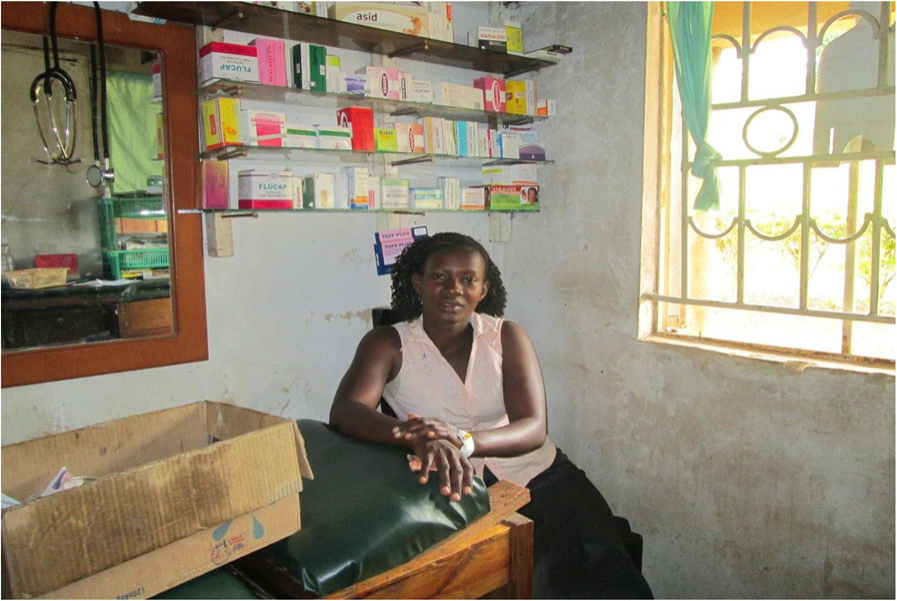
A female CHW seeking health care at a local health facility

During the monthly discussions, CHWs revealed that they needed to be exemplary in all aspects of their work including observance of proper sanitation and hygiene practices in their homesteads, seeking health care from health facilities when sick (Fig. 1), and proper use of health commodities including those provided by the government such as mosquito nets.

### Ethical responsibility in patient care

**Fig. 2:**
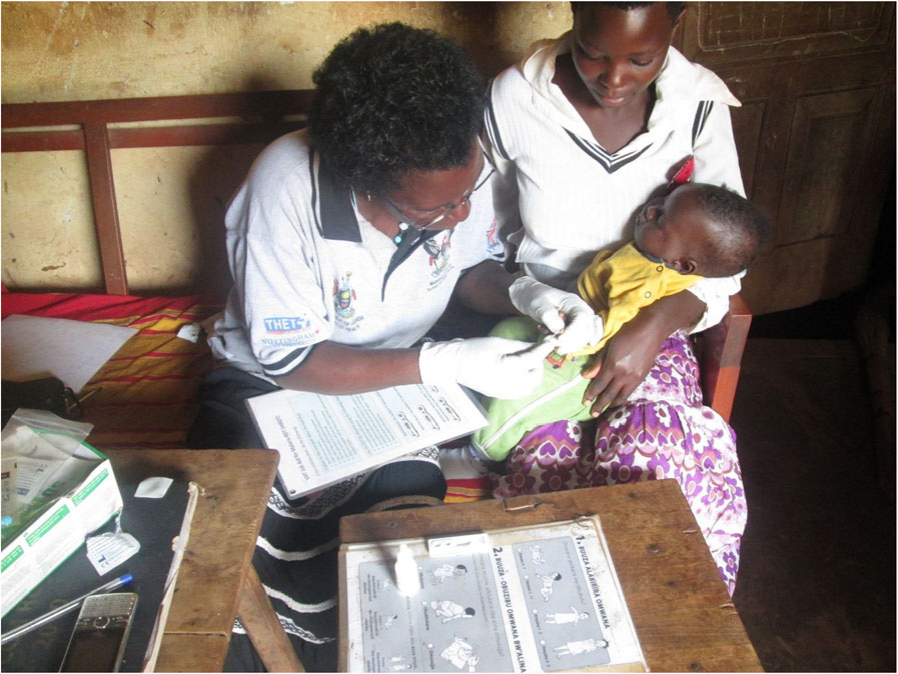
A female CHW drawing a blood sample from a sick child for malaria diagnosis before treatment while consulting her job aids

Some CHWs (two out of the four per village) are engaged in integrated community case management (iCCM) of malaria, diarrhoea and pneumonia among children under 5 years of age. These CHWs noted that ethical principles in carrying out such clinical work was very important. Indeed, the CHWs pointed out issues ranging from consulting job aids to understand the treatment procedure, and carrying out proper diagnosis of patients before administering drugs (Fig. 2). In addition, the CHWs noted that they ensured to treat only those children they could manage, and referred complicated cases to health facilities. CHWs who were not involved in iCCM would refer cases to their colleagues who manage childhood illnesses.

*“Although we treat children, we are not supposed to handle those above five years of age. If a sick child of six or seven years is brought to us, we can only refer them to a health facility for diagnosis and treatment. It is ethical for us community health workers to follow and stick to guidelines provided to us.*” CHW 10, male

In case of emergencies such as disease outbreaks, severely sick children, and women experiencing labour pains, CHWs responded very fast by offering first aid, rushing the sick to the health facility, or offering any other assistance within their means as the first contact of the health system at community level.

*“I found a child who had diarrhoea in the village but people were afraid of her saying she could be having cholera. I gave her first aid before asking the parents to take her to the health facility. The parents were still afraid of the child in fear of contracting the disease, and I decided to rush her to the health facility using my motorcycle.”* CHW 9, male

### Maintaining confidentiality while handling clients

**Fig. 3:**
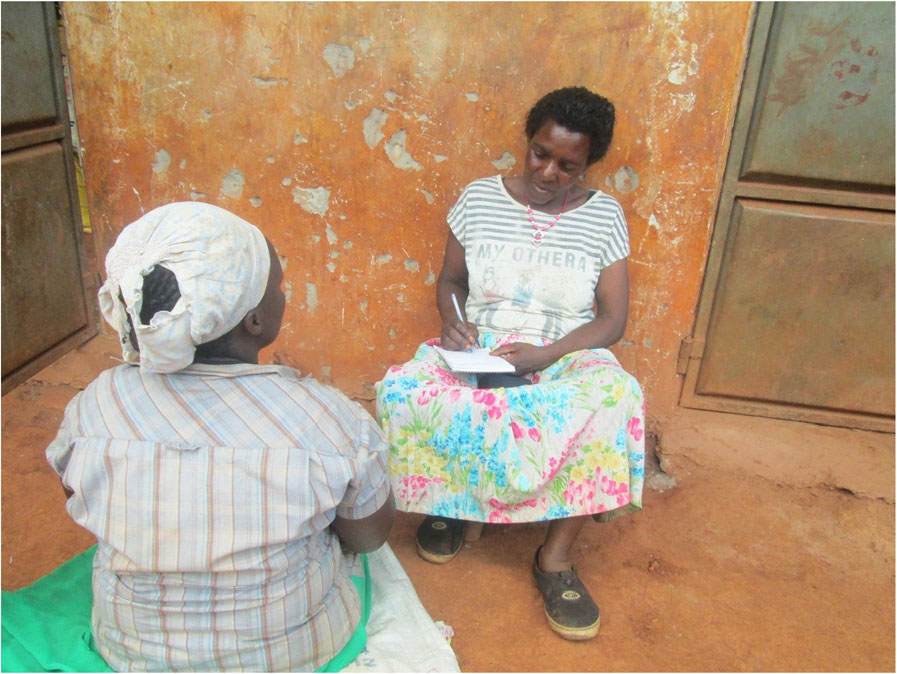
A CHW during a consultation with a pregnant woman within a setting that ensured privacy

People often share with CHWs their health problems in order to get treatment, advice, referrals or counselling. CHWs revealed that it was very important to maintain confidentiality while dealing with clients in the community. CHWs also said that while treating or interacting with clients, they usually held one-on-one discussion in places that provided privacy (Fig. 3).

*“An HIV positive man came to consult me claiming how his antiretroviral medicine had affected his vision. Although I am now aware of his HIV status, I can never disclose it to anyone.*” CHW 8, female

### Respect for persons and communities

CHWs noted that it was very important to respect the communities they served including their cultures, norms and traditions. The CHWs said this concern could be demonstrated through commitment in serving the community, engaging in community activities, and attending community functions including religious events. The CHWs also stated that all community members should be respected and treated without any discrimination based on age, gender, ethnicity, religion or otherwise.

*“I go to church every Sunday. Participating in such activities with other community members helps in building trust amongst us. In fact, I am often recognised as a dedicated community member which makes my work as a community health worker easier.”* CHW 1, male

CHWs noted that while carrying out their work, self-identification was very important. The CHWs acknowledged that they ensured identification through wearing provided uniforms, and introducing themselves to the community during their course of work.

*“That woman asked me that, ‘who are you to ask me such questions?’ I told her that I am a community health worker just as my t-shirt suggests. She then asked me where I come from and I told her that I am a resident of the village. Wearing my t-shirt at the time helped very much in this situation.*” CHW 9, male

### Enhancing knowledge and skills

**Fig. 4:**
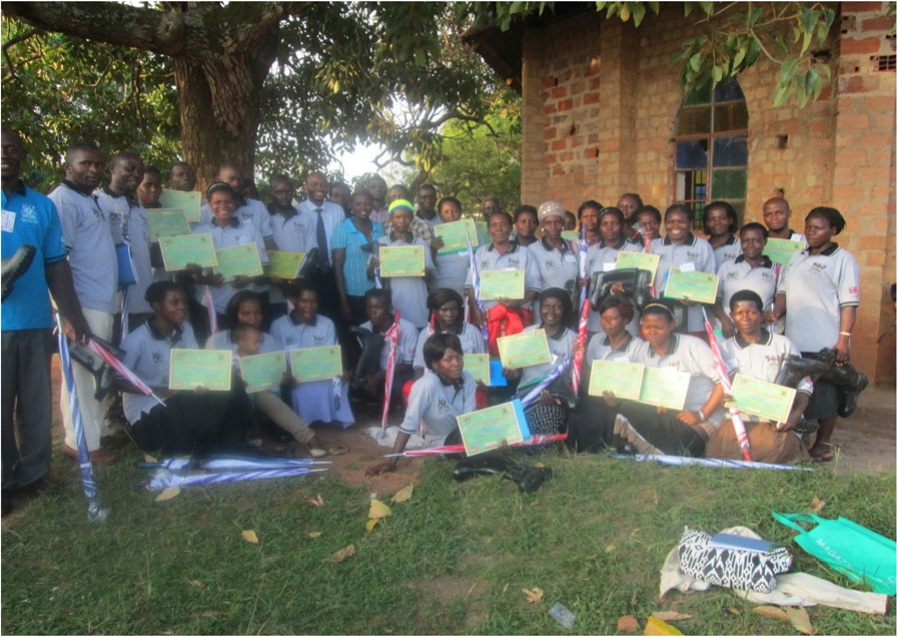
CHWs displaying their certificates and non-financial incentives they had received after attending a training

CHWs revealed that it was vital for them to continuously enhance their knowledge and skills at any given opportunity so as to be able to provide high quality, ethical service to individuals, families, and their communities. The CHWs therefore endeavoured to attend trainings, workshops and seminars to learn more about health issues, and enhance their knowledge and skills whenever the opportunity arose (Fig. 4). In addition, the CHWs also accessed health information through mass media channels such as radio, television, newspapers and phones so that they were up-to-date with pertinent health issues within their community and country.

“*The radio gives us information and knowledge on a number of health issues. As community health workers, it is good practice that we continue to seek the right information and learn more about health since knowledge on these issues keeps changing.*” CHW 10, male

CHWs also noted that they enhanced their knowledge and skills through working together as teams, and sharing what they learnt with colleagues who may not have attended some trainings. The CHWs also continuously enhanced their knowledge and skills through sharing it with other community members including students.

*“Whenever I went to visit homes in the community, hand washing facilities were non-existent. When I asked fellow community health workers about it, they told me that they did not know how to make these facilities. I then started training my colleagues on making hand washing facilities because I realised that it would be easier to reach the entire community. Through that approach of teamwork, we can support each other for the betterment of our community.”* CHW 1, male

### Challenges in the work of CHWs that undermine adherence to ethics principles

Although CHWs were very keen to maintain and ensure that ethical principles were observed in their work, they faced a number of challenges. These challenges included: uncooperative community members; minimal avenues for knowledge enhancement; lack of necessary supplies such as medicines, rapid diagnostic tests (RDTs) and reference materials; and guidelines being available in English yet majority could read the local language better.

*“I diagnosed a sick child for malaria using a rapid diagnostic kit. However, I did not have any malaria drugs supplied by the Ministry of Health to CHWs hence I could not offer her medication which was not a good thing. Lack of essential medicines therefore negatively affects our work as community health workers.”* CHW 10, male

There were also concerns of low levels of commitment from some CHWs especially the males in performing their roles. These concerns were mainly attributed to more involvement of male CHWs in income generating activities to support their families. Female CHWs were also engaged in domestic chores which at times limited their availability for community health work.

*“Although we carry out our duties as community health workers, we also need to look for ways of earning an income to sustain our families. In this village, some community health workers work in stone quarries to raise income. After such a day’s work, they are usually too tired to engage in community health duties. In addition, many times female community health workers have to do domestic work including laundry, cooking, fetching water among other house cores which limits their time to support their communities.”* CHW 10, male

## Discussion

This study explored the important issue of ethical practice through the eyes of CHWs using photovoice. The roles of CHWs necessitate a strict observance of ethical principles for their benefit and that of the communities they serve. Moreover, when CHWs observe a high moral standard during their work, there is assurance to the community that those with the responsibility to serve them are doing the right thing which further builds trust. It was therefore significant that CHWs were aware of the importance of ethical principles in their work and took steps to observe ethical principles amidst the challenges they faced. The key ethical values CHWs espoused included maintaining professional integrity and abiding by ethical principles of practice including in patient care, maintaining confidentiality while handling clients, respect for persons and communities, and enhancing their knowledge and skills which are all in line with the code of ethics developed by the American Association of Community Health Workers [19]. The CHWs also highlighted minimal avenues for knowledge enhancement, shortage of supplies, guidelines being written in English, and ‘uncooperative community members’ as working to undermine their ethical practice.

During the course of carrying out their duties, CHWs expressed the need to maintain professional integrity and abide by ethical principles of practice. This concern included embracing values of honesty and truthfulness in addition to being committed to serving their communities. Honesty and truthfulness are key in any health related work, and enshrined in many of the professional codes of ethics [20, 21]. In communities where health workers are few and health facilities distant, CHWs are usually faced with health challenges that are beyond their scope of practice. In fact, CHW roles have increasingly become more curative, and their boundaries not well defined in many countries [2, 22]. This situation has the potential of creating ambiguity and thus as a starting point, CHW roles and scope of work, to which they ought to abide, should be clearly defined. At the same time, previous studies have shown that CHWs struggle to balance their health work responsibilities which are typically voluntary in most countries including Uganda with other economic activities which are a source of their livelihood [2, 23, 24] which was also established in our study. For sustainability and effectiveness of CHW programmes, it is important that governments put in place strong remuneration mechanisms that recognise the contribution of CHWs to the health system in line with the World Health Organization guidelines to optimize CHW programmes [25]. Such mechanisms would support CHWs in observing integrity to their work as they carry out their roles for example by committing more time.

In their role, CHWs are involved in caring for patients usually after receiving skills through training to treat children under 5 years against malaria, pneumonia and diarrhoea [26]. Besides training, CHWs should be provided with job aids that they can continuously consult while caring for patients, in a language that is easy for them to understand. Patient care also comes with the responsibility of referral, and CHWs should be made to fully understand available referral systems and protocols. In patient care, a key ethical principle identified in the study was maintaining confidentiality which has often been reported as a challenge in community work [27]. This challenge is especially important because community members are based in communities by definition and can receive significant confidential information. Breaching that confidentiality could hinder the community from seeking care from them in addition to causing conflict [28, 29]. Besides, some health conditions could also be associated with stigma in the community if disclosed further justifying the need for CHWs to uphold their confidentiality obligation.

Respect has been acknowledged as one of the ethical virtues of community health work [30] and its importance highlighted in previous research [31, 32]. In their proposed ethical framework for CHWs and related institutions, Stone and Parham (2007) rank respect which they refer to as ‘equal and substantial respect’ as the most fundamental principle in addition to justice and care [33]. By being respectful, CHWs are able to treat their community members’ needs as significant and provide them care that is sensitive to their context and personal factors. In addition, regarding justice, CHWs are expected not to treat people differently based on their characteristics such as race, religion, ethnicity and culture as established in our study.

In our study, CHWs noted the need to continuously build their capacity to serve their communities better. Indeed, CHWs should keep abreast with latest developments regarding their work by enhancing their knowledge and skills through regular training opportunities. Through refresher trainings, CHWs build competency, another key ethical dimension of CHW work [33], which is recognised in other professional codes of ethics [20, 21]. Unfortunately, for many CHW programmes especially in sub-Saharan Africa, training gaps and the lack of regular capacity building initiatives have been reported to affect their efficiency and effectiveness [2, 23, 24]. Other stakeholders such as health practitioners and community members have also reportedly questioned the competency of CHWs to carry out their roles [28, 29]. Moreover, many training curricula do not emphasize the importance of ethics in CHW work neither do many country programmes in sub-Saharan Africa have an established CHWs code of ethics. Policy makers should establish such codes of ethics to which CHWs should abide, and incorporate them in future training programmes together with avenues for routine training, support processes, and career development.

CHWs reportedly encountered many challenges in their work, including uncooperative community members, minimal avenues for knowledge enhancement, lack of necessary supplies and equipment such as medicines and rapid diagnostic tests, and reference materials / guidelines being available in English yet majority were more fluent in the local language. These challenges have been well documented by CHW programmes around the world not merely as ethical challenges but also concerns that hinder their performance and retention [23, 24, 34–36]. With this realisation, it is imperative that measures are in built within CHW programmes to address these challenges right from their design. CHWs should also be supported to deal with some of these ethical dilemmas partly through regular support supervision by their superiors including health practitioners. The community also ought to be sensitised about the roles of CHWs and their contribution to address health problems, as well as to hold them accountable to carrying out their responsibilities diligently. Importantly, funding for CHW programmes should be increased now more than ever. Indeed, CHWs should be moved away from the fringes of the health system where they are undefined and unsupported [37] to being fully institutionalised within the health system to be able to deal with such ethical and performance related challenges to achieve their potential [38].

Using photovoice, this study enabled CHWs to record and reflect on ethical concerns and challenges in their work. In addition, CHWs had a critical dialogue on the identified issues through group discussions of photographs and community disseminations where the findings reached policy makers hence achieving the goals of photovoice [39]. Moreover, a key strength of photovoice is its ability to allow people with limited power such as CHWs, to capture aspects of their environment and experiences, and share them with others [17]. The experiences of applying this methodology in this study are similar to those that we have previously reported [16]. This study was carried out in a rural context similar to most areas in which CHWs operate within Uganda. In addition, an equal number of male and female CHWs from different villages were involved which are strengths of the study.

## Conclusions

Through photovoice, we found that CHWs were aware of the importance of observing ethical principles in their work, and strived to uphold ethical values such as maintaining professional integrity, ensuring ethical responsibility in patient care, maintaining patient confidentiality, and respect for persons and communities. However, CHWs were faced with challenges that impacted on their work including uncooperative community members, minimal avenues for knowledge enhancement, and lack of supplies that undermined their ethical practice. Ministries of health and other stakeholders supporting CHW programmes should create an enabling environment for them to uphold ethical principles through incorporating it in their training, establishing an ethical code of practice, and providing necessary requirements for their work.

## Data Availability

Data and materials of the project are available from the corresponding author on reasonable request.

## Abbreviations

CHW: Community health worker
iCCM: Integrated community case management of childhood illnesses
LMIC: Low- and middle-income country
VHT: Village health team
